# Oral Hygiene Practices and Fluoride Toothpaste Use in Children Under 3 Years Old in Lima, Peru: A Cross‐Sectional Study

**DOI:** 10.1155/ijod/7483990

**Published:** 2026-10-07

**Authors:** Rosa J. Muñoz-Nuñez, Josselyn R. Ramirez-Escapa, Ailín Cabrera-Matta

**Affiliations:** ^1^ Department of Dentistry for Children and Adolescents Universidad Peruana Cayetano Heredia Lima Peru upch.edu.pe; ^2^ Program Pediatric Dentistry Universidad Peruana Cayetano Heredia Lima Peru upch.edu.pe

**Keywords:** child preschool, dental care for children, fluorides, infant, maternal-child health services, toothpastes, topical

## Abstract

**Aims and Background:**

Early childhood caries (ECC) is the most common noncommunicable disease in children, according to the World Health Organization (WHO). The daily use of fluoride toothpaste is the most effective strategy for caries prevention and control. This study aims to determine the oral hygiene practices and use of fluoride toothpaste in children under 3 years old.

**Materials and Methods:**

Using a questionnaire about oral hygiene habits, this cross‐sectional study surveyed 502 parents and caregivers of children under 3 years old during pediatric check‐ups at a hospital in Lima, Peru. Descriptive statistics were used to summarize the data; absolute and relative frequencies were calculated for categorical variables. Quantitative variables were assessed using graphical methods, descriptive statistics, and summarized as medians and interquartile ranges due to their nonnormal distribution. Fisher’s exact test was applied to assess the association between the toothpaste type and the child’s age, with a significance level of 0.05.

**Results:**

Some form of oral hygiene was reported in 79.5% (*n* = 399) of children. While 27.5% (*n* = 138) of parents/caregivers brushed their children’s teeth with toothpaste at least twice daily. Only 19.3% (*n* = 97) of them used a toothpaste with at least 1000 ppm F on a daily basis. The most commonly used amount of toothpaste was 1/8 of the brush head (45.5%). The most common sources of recommendation regarding the type of toothpaste were dentists, physicians, and pharmacy staff.

**Conclusion:**

Despite current global recommendations, only a small proportion of children under 3 years of age used fluoride toothpaste containing at least 1000 ppm, and age‐related differences were observed. These findings highlight the need for pediatric dentists to strengthen parental education and to support the training of both dental and nondental health professionals in evidence‐based oral hygiene practices in early childhood.

## 1. Introduction

Dental caries is currently defined as a “biofilm‐mediated, sugar‐driven, multifactorial, and dynamic disease that results in the phasic demineralization and remineralization of dental hard tissues” [1]. In children under 6 years of age, this disease is called early childhood caries (ECC) [2], which globally represents the most common noncommunicable disease in this age group, according to a report published by the World Health Organization (WHO) in 2020 [3]. In Peru, the national ECC prevalence is 76.2% in children between three and 5 years old [4]. Due to its complexity, a combination of community, professional, and individual strategies is needed for prevention and control of ECC [2].

Within the interventions against ECC, the use of fluoride toothpaste is one of the most effective methods [1, 5, 6]. For that reason, toothbrushing twice daily with fluoride toothpaste with at least 1000 ppm and using an age‐appropriate amount is a public health intervention recommended globally [2, 3]. The use of nonfluoride and low‐fluoride toothpastes does not provide the desired anticaries effect and is rather considered a risk factor [1]. It should be noted that, to ensure proper ECC control, WHO, the International Association of Pediatric Dentistry (IAPD), and other scientific organizations recommend tooth brushing with fluoride toothpaste since the eruption of the first tooth [2, 3, 7].

Despite these evidence‐based recommendations regarding the use of fluoride toothpaste, there is still a large proportion of parents/caregivers of preschool children who are not following them [8–12]. Parents and primary caregivers have a great impact on the oral health practices of preschool children as they help the child to establish and maintain these habits [13, 14]. Studies on parental knowledge and practices regarding toothpaste use reported that parents/caregivers lacked information [8–12].

In Peru, community water fluoridation is not implemented as a public health measure, and information regarding naturally occurring fluoride levels in drinking water is limited. Although salt fluoridation has been established through national regulations, the evidence regarding its implementation and monitoring at the population level remains limited [4]. In this context, fluoride toothpaste use represents a key preventive measure against dental caries, as recommended by the Clinical Practice Guideline of the Peruvian Ministry of Health [15]. Therefore, the main purpose of this study was to determine the proportion of children under 3 years of age using a fluoride toothpaste with at least 1000 ppm. Additionally, we sought to describe toothbrushing and other oral hygiene practices in this age group and to determine the type of toothpaste used according to age.

## 2. Methods

### 2.1. Study Design and Population

This cross‐sectional study was conducted at the pediatric check‐up service for children under 6 years of age at a public maternal and child hospital in Lima, Peru. The participants were parents or caregivers of children under 3 years old who sought medical care between November 2019 and March 2020. Children were eligible if they had at least one erupted tooth. Parents or caregivers who were not living with the child were excluded.

### 2.2. Sample Size

The sample size was calculated using a 95% confidence level, 3.5% precision, an anticipated prevalence of 20% of children under 3 years old using toothpaste containing at least 1000 ppm fluoride, and an infinite population assumption. The estimated sample size was 502 parent/caregiver–child pairs. To achieve this sample size, consecutive sampling was employed.

### 2.3. Data Collection

We used a structured questionnaire with close‐ended questions to evaluate fluoride toothpaste use and oral hygiene practices in children under 3 years old. The validity of the questionnaire was independently reviewed by three pediatric dentistry professors, with at least 15 years of experience. They provided feedback and suggestions for improvement. After incorporating their recommendations, a pilot study was conducted at the hospital with 50 parents/caregivers (10% of the calculated sample size). Based on the pilot results, the questionnaire was revised to improve its clarity before data collection. The questionnaire included two sections: demographic characteristics and oral hygiene practices. In the latter, we included brushing habits, the age at which toothbrushing was initiated, the type of toothpaste currently used, the amount of toothpaste used, the frequency of toothbrushing, and the sources of recommendations for these practices. The questionnaire was administered in the waiting room of the pediatric service to the parents/caregivers at the end of the child’s pediatric check‐up. Two trained researchers administered the questionnaire through face‐to‐face interviews.

### 2.4. Statistical Analysis

Descriptive analysis was carried out in accordance with the main objective of the study. Absolute and relative frequencies were calculated for qualitative variables. The distribution of quantitative variables was assessed using graphical methods and descriptive statistics, including skewness and kurtosis. As the quantitative variables showed a nonnormal distribution, they were summarized using medians and interquartile ranges. For the analysis of the type of toothpaste according to the child’s age, we used Fisher’s exact test with a significance level of 0.05. Stata version 16 was used for all calculations.

### 2.5. Ethical Considerations

This study was approved by the Institutional Ethics Committee of Universidad Peruana Cayetano Heredia (104189) and the Ethics Committee of San Bartolome National Hospital (14365–19). Each parent/caregiver gave written informed consent prior to enrollment.

## 3. Results

A total of 502 parents/caregivers of children under 3 years of age participated in the study. Out of 563 invited individuals, the response rate was 89.2% (*n* = 502). The main reason for declining to take part was a lack of time during the hospital visit. Participant demographics are summarized in Table 1. Most children were between 12 and 23 months old, and mothers were the primary respondents (81.7%). Almost half of the children had no siblings.

**Table 1:** Characteristics of parents/caregivers and their children attending a pediatric check‐up at a public hospital in Lima, 2020 (*n* = 502).

Variables	*n* (%)
Child’s age (months)	
4–11	189 (37.7)
12–23	213 (42.4)
24–36	100 (19.9)
Child’s sex	
Male	265 (52.8)
Female	237 (47.2)
Parent/caregiver’s age (years)^a^	31 (27–37)
Parent/caregiver’s sex	
Male	64 (12.8)
Female	438 (87.3)
Parent/caregiver’s relationship	
Mother	410 (81.7)
Father	54 (10.8)
Grandparent	22 (4.4)
Other relative	16 (3.2)
Number of brothers and sisters^a^	1 (0–1)

^a^Median (interquartile range).

Table 2 presents oral hygiene practices. Most of the participants, 79.5% (*n* = 399), reported performing some form of oral hygiene on their children, while 43.2% (*n* = 216) reported using a toothbrush. Additionally, 13 parents/caregivers (6.0%) used a toothbrush without toothpaste. Out of the 224 parents/caregivers who reported using toothpaste, 21 applied it with a baby finger toothbrush or gauze, while the remaining 203 (40.4%) used it together with a regular toothbrush. Daily brushing with toothpaste was reported by approximately one‐third of participants, while less than one‐third reported brushing their children’s teeth at least twice a day.

**Table 2:** Oral hygiene practices reported by parents/caregivers of children under 3 years of age attending a pediatric check‐up at a public hospital in Lima, 2020 (*n* = 502).

Practices		*n* (%)
Teeth cleaning		399 (79.5)
Aids used for oral hygiene		
Toothbrush		216 (43.2)
Gauze		120 (23.9)
Finger toothbrush		44 (8.8)
Cloth		16 (3.2)
Other implements		3 (0.6)
Toothpaste use		224 (44.6)
Combined use of toothbrush and toothpaste		203 (40.4)
Daily brushing with toothpaste		178 (35.5)
Brushing frequency with toothpaste (*n* = 178)		
Once a day		38 (21.4)
Twice a day		102 (57.3)
More than twice a day		37 (20.8)
Do not know/do not remember		1 (0.6)
Daily brushing with toothpaste at least twice a day		138 (27.5)

Table 3 summarizes the reported practices regarding toothpaste use. Among all respondents, 19.3% (*n* = 97) reported using toothpaste with a fluoride concentration of at least 1000 ppm. The primary source of recommendation regarding toothpaste type was the dentist. The bivariate analysis of toothpaste by children’s age group (Figure 1) indicated that at least one of the age groups exhibited a significantly different distribution compared to the other groups (*p* = 0.009). The difference was most apparent in the 24‐ to 36‐month age group, where the majority of children were found to use toothpaste with a fluoride concentration of 1000–1500 ppm.

**Figure 1:**
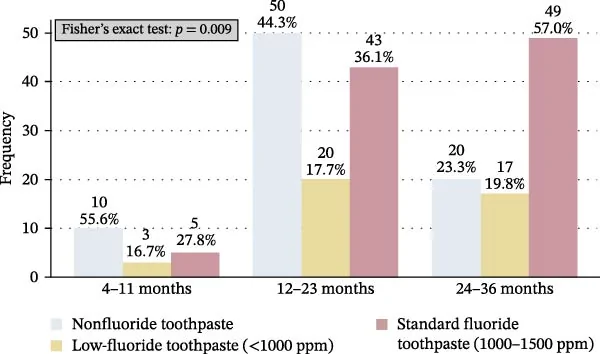
Distribution of toothpaste type by age among children attending a pediatric check‐up at a public hospital, as reported by parents/caregivers in Lima, 2020 (*n* = 217).

**Table 3:** Practices regarding toothpaste use among children under 3 years of age attending a pediatric check‐up at a public hospital in Lima, as reported by parents/caregivers, 2020.

Practices	*n* (%)
Toothpaste use (*n* = 502)	224 (44.6)
Type of toothpaste used (*n* = 224)	
Nonfluoride	80 (35.7)
Low fluoride (<1000 ppm)	40 (17.9)
Standard pediatric (1000–1100 ppm)	86 (38.4)
Standard for adults (>1100–1500 ppm)	11 (4.9)
Do not know/do not remember	7 (3.1)
Use of toothpaste with ≥1000 ppm fluoride (*n* = 502)	97 (19.3)
Source of recommendation for toothpaste type (*n* = 224)	
Dentist	67 (29.9)
Physician	48 (21.4)
Pharmacy staff	48 (21.4)
Relatives/friends	28 (12.5)
Other healthcare professionals	14 (6.3)
Traditional media	8 (3.6)
Product labeling	5 (2.2)
Others	6 (2.7)
Age of initiation of toothbrushing with toothpaste (*n* = 203)	
<12 months	70 (34.5)
12–23 months	112 (55.2)
24–35 months	21 (10.4)
Daily toothbrushing with toothpaste (*n* = 502)	178 (35.5)
Nighttime toothbrushing with toothpaste (*n* = 178)	146 (82.0)
Amount of toothpaste used (*n* = 178)	
1/8 of the toothbrush head length	81 (45.5)
1/4 of the toothbrush head length	60 (33.7)
1/2 of the toothbrush head length	27 (15.2)
>1/2 of the toothbrush head length	10 (5.6)
Source of recommendation for the amount of toothpaste (*n* = 178)	
Dentist	64 (36.0)
Physician	33 (18.5)
Relatives/friends	23 (12.9)
Pharmacy staff	16 (9.0)
Self‐selected amount	15 (8.4)
Traditional media	12 (6.7)
Nurse	7 (3.9)
Social media	7 (3.9)
Product labeling	1 (0.6)
Main reason for toothpaste brand selection (*n* = 178)	
External recommendation	104 (58.4)
Trusted brand	25 (14.0)
Child‐preferred flavor	14 (7.9)
Age recommendation on product labeling	10 (5.6)
Nonfluoride	8 (4.5)
Only option available at time of purchase	5 (2.8)
Price	4 (2.3)
Low‐fluoride concentration	3 (1.7)
Other	5 (2.8)

## 4. Discussion

We found that 79.5% (*n* = 399) of parents/caregivers of children under 36 months with at least one erupted tooth performed some form of oral hygiene for their children, yet only 44.6% (*n* = 224) used toothpaste. Fewer than half used a fluoride toothpaste with at least 1000 ppm F when brushing their children’s teeth; this group represented 19.3% (*n* = 97) of the total sample. Interestingly, the primary source of recommendations regarding the type and amount of toothpaste was the dentist.

Global and regional organizations have issued evidence‐based recommendations to control ECC, including the use of fluoride toothpaste with at least 1000 ppm F in an appropriate amount for the child’s age, at least twice a day since the eruption of the first tooth, and scheduling the first visit to the dentist before the first birthday [3, 4, 7, 16, 17]. In Peru, these recommendations are also outlined in two documents issued by the Peruvian Ministry of Health [4, 15, 18].

Regarding brushing frequency, our study found that among parents who reported brushing their children’s teeth daily (*n* = 178), 57.3% (*n* = 102) reported doing so twice a day. Similar results were reported in a 2020 study conducted in Chicago, USA, involving 148 participants, in which 50% reported brushing their children’s teeth twice a day [10] and in a 2016 study conducted in Teresina, Brazil, involving 148 participants, in which 50.67% reported the same practice [19]. Additionally, a study conducted in Switzerland in 2023, involving 200 children under 3 years of age, found that two‐thirds of children under 3 years of age brushed their teeth at least twice a day [20]. In contrast, when considering the entire sample in our study (*n* = 502), only 27.5% (*n* = 138) brushed their children’s teeth with toothpaste at least twice a day. This result differs considerably from a study in the USA in 2020, which included 148 children and reported that 59.5% of children under 3 years of age followed this practice [10]. This discrepancy is probably due to differences between the USA and Peruvian public health systems.

Our results show that only 19.3% (*n* = 97) of all parents/caregivers used toothpaste with a minimum of 1000 ppm F for their children. This situation is likely attributable to the fact that, in Peru, all children’s toothpastes containing at least 1000 ppm F are labeled (both in their external and internal packaging) as intended for children aged 2 years and older [21]. Results similar to ours have been reported in other countries. A 2016 study carried out in New Zealand, involving 984 children from 4 months to 4 years old, found that 19% of parents used toothpastes with at least 1000 ppm F when brushing their children’s teeth [8]. The researchers suggested that these results may be due to the lack of participation of nondental healthcare professionals, who have contact with children at a younger age, in oral health policies. In contrast to our results, a Norwegian study found that all children between 1 and 5 years old used a toothpaste with a minimum of 1000 ppm F. This might be because all toothpastes for children contain 1000 ppm F there, and Norwegian health authorities have issued oral health recommendations [9]. In Peru, a study published in 2018 used a national survey database of 41,330 records and found that 28.5% of children between one and 5 years old used a toothpaste with a minimum of 1000 ppm F [22]. This percentage is higher than ours, and the discrepancy could be attributed to the differences in the age ranges of the samples.

We found that dentists (29.9%, *n* = 67) were the primary source of recommendation for the type of toothpaste, followed by physicians (21.4%, *n* = 48) and pharmacy staff (21.4%, *n* = 48). Despite this, the proportion of children using toothpaste with an adequate fluoride concentration was low, suggesting that recommendations from healthcare professionals do not necessarily translate into appropriate toothpaste use. A study conducted in England implemented a comprehensive oral health program focused on professional education and the engagement of primary care providers, which reduced the experience of dental caries in children by 8% over a 3‐year period [23]. These results highlight the importance of coordinated actions between primary care providers and the integration of oral health education into the training of health professionals who work with young children. Similarly, a study in South Australia evaluated the sources of information available to parents regarding infant oral health and reported that the most frequently cited sources were brochures, child health nurses, other caregivers (such as playgroup mothers), and dentists [24]. Mothers who consulted multiple sources—particularly dental professionals—were more likely to follow positive oral hygiene practices; however, no association was observed between the sources of information and toothpaste use in infants [24]. This suggests that although access to information may improve general oral hygiene practices, specific guidance on fluoride toothpaste use may still be insufficient, highlighting the need for improved training of nondental healthcare professionals who advise parents.

Our findings show that 82.0% (*n* = 146) of parents/caregivers who reported daily brushing with toothpaste also did so at night. We hypothesize that this high percentage is owing to the fact that parents, because of their work responsibilities, have more time at night, making nighttime toothbrushing more feasible. Huebner and Riedy [25] conducted a study in a rural area of Washington and found that parents’ lack of time in the morning was considered a limitation that affects regular toothbrushing of their children under 5 years of age. Similarly, a study by Berzinski et al. [13] revealed that parents have greater difficulty carrying out a morning toothbrushing routine. In contrast, Trubey et al. [26] found that the majority of parents brushed more frequently in the morning than at night. They associated this finding with the tendency observed in parents to focus on the esthetic benefits of daytime toothbrushing in the short term compared to those of evening brushing.

To reduce side effects in children, fluoride toothpaste should be used in an age‐appropriate amount [2, 3, 7]. We found that the most frequent quantity of toothpaste reported was 1/8 of the brush head length (45.5%, *n* = 81). A study involving children between 6 months and 6 years old in Saudi Arabia found that the most used amount was a pea‐size quantity of 42.2% [27]. This discrepancy may be attributed to the differing age ranges of children in the aforementioned studies. Similar to our findings of a minimal amount of toothpaste, Avenetti [10] reported that, in children under 3 years of age, the most commonly used amount was a “smear” in Chicago, USA (55.7%).

The main reason for choosing a toothpaste can be highly diverse. Some reported reasons behind this decision are trust in the brand, family routines, and flavor [8]. In our study, the most frequent reason was the recommendation of another person (58.4%, *n* = 104), most of whom were health professionals. However, as mentioned before, we found a high proportion of parents/caregivers who do not follow an evidence‐based oral hygiene routine with their children. Given this situation, we consider it necessary to improve the knowledge of toothpaste use and child oral hygiene among general dentists and nondental health professionals, especially those who have early contact with young children and their families.

In the analysis of the type of toothpaste used according to age, we found that 57.0% (*n* = 49) of the group between 24 and 36 months old used toothpastes with at least 1000 ppm F. Children younger than 24 months old used a nonfluoride toothpaste. This could be due to the fact that in Peru, commercially available toothpastes for children younger than 2 years of age do not contain fluoride [21, 28]. Moreover, the Peruvian regulation regarding the standards for adding fluoride in toothpastes stipulates that the fluoride concentration must be between 250 and 550 ppm for children under 6 years of age [18]. This norm, published in 2001, needs to be updated because it probably contributes to the existence of toothpastes with inadequate fluoride concentrations.

We should note that our sample was limited to a single public hospital in Lima; therefore, the findings should be interpreted with caution as they likely do not represent all users of the Peruvian health system, both public and private. Nevertheless, our results describe toothpaste use practices in an important population group—children attending the pediatric check‐up service. Although the sample was not drawn from all metropolitan Lima, our findings may reflect patterns occurring in this setting. One possible limitation of the study is that there could be a difference between the type and amount of toothpaste reported by the parent/caregiver and what they actually used, as reported by Siqueira in 2019 [29]. However, self‐report data from surveys are used by the scientific community because they are an important approximation and contribute to the formulation of hypotheses and research questions for future studies. There are few publications on oral hygiene practices and the proper use of fluoride toothpastes in children under 3 years old. It has been reported that the establishment of hygiene behavior at an early age lasts over time during the preschool stage [9]. Therefore, it is very important to carry out studies that analyze these practices, as exemplified by the present study. Future studies should investigate factors influencing the choice of toothpaste for children and how oral hygiene practices change over time during early childhood.

## 5. Conclusion

Most parents/caregivers of children under 3 years of age attending the pediatric check‐up service of a public hospital reported oral hygiene practices that did not consistently align with evidence‐based recommendations for fluoride toothpaste use. Only a limited proportion of children used toothpaste containing at least 1000 ppm fluoride, and toothpaste amounts exceeding age‐based recommendations were frequently reported. Dentists, physicians, and pharmacy staff were the most frequently reported sources of recommendations regarding toothpaste selection. Differences according to age were observed, with children aged 24–36 months more likely to use toothpaste with at least 1000 ppm fluoride than younger children. These findings highlight the importance of oral health education for parents/caregivers and training of nondental health professionals involved in ECC.

**Nomenclature**
ECC: Early childhood caries
WHO: World Health Organization
IAPD: International Association of Pediatric Dentistry.

## Author Contributions

**Ailín Cabrera-Matta**: conceptualization, formal analysis, funding acquisition, investigation, methodology, project administration, supervision, validation, visualization, writing – original draft, writing – review and editing. **Rosa J. Muñoz-Nuñez**: data curation, formal analysis, funding acquisition, investigation, methodology, resources, software, visualization, writing – original draft, writing – review and editing. **Josselyn R. Ramirez-Escapa**: data curation, formal analysis, funding acquisition, investigation, methodology, software, visualization, writing – original draft.

## Funding

This study received funding from the Institutional Research Support Fund of the Faculty of Stomatology, Universidad Peruana Cayetano Heredia. Upon acceptance, funding for publication will be sought from the Faculty of Stomatology of Universidad Peruana Cayetano Heredia.

## Disclosure

All authors have read and approved the final version of the manuscript. Ailín Cabrera‐Matta had full access to all of the data in this study and takes complete responsibility for the integrity of the data and the accuracy of the data analysis. No external support, contributions, or assistance were received that warrant recognition.

## Conflicts of Interest

The authors declare no conflicts of interest.

## Data Availability

The data that support the findings of this study are available upon request from the corresponding author. The data are not publicly available due to privacy or ethical restrictions.
